# Supplementary material for: Statistical Basis for Predicting Technological Progress
Source: PLoS One. 2013 Feb 28;8(2):e52669. doi: 10.1371/journal.pone.0052669 (PMC3585312; doi:10.1371/journal.pone.0052669)
Supplement: File S1 — Supporting Information (PDF) [file pone.0052669.s001.pdf]

Statistical Basis for Predicting Technological Progress
Supporting Information

Béla Nagy, J. Doyne Farmer, Quan Minh Bui, Jessika E. Trancik

January 21, 2013

Contents

1 Data set **1**

2 Exponential increase of production **4**

3 Hindcasting results **4**

4 Error model **15**

4.1 Choosing the response 22
4.2 Modeling the response 22
4.3 Statistical model 23
4.4 Intercept and slope parameter estimates 23

5 Extrapolation method **23**

1 Data set

This section contains four tables for the 62 technologies used in the paper, divided into four industry groups: Chemical, Hardware, Energy, and Other. In each row, after the name of a particular product, a contiguous time period is specified for which data was available, followed by the number of data points in the resulting yearly time series. Then the parameter estimates g, m, and w are given, followed by the corresponding cumulative production volume doubling times, unit price halving times, and progress ratios, respectively.

To facilitate our research and the exchange of information, we built a data repository called the Performance Curve Database at `http://pcdb.santafe.edu/`. References for all the data sources and the data for the 62 technologies analyzed in this paper are available in the online

Chemical Industry	*time period*	*data points*	*g*	*m*	*w*	*doubling time*	*halving time*	*progress ratio*
AcrylicFiber [1]	1960 - 1972	13	0.076	0.045	0.58	4.0	6.8	0.67
Acrylonitrile [1]	1959 - 1972	14	0.077	0.033	0.43	3.9	9.1	0.74
Aluminum [1]	1956 - 1972	17	0.035	0.004	0.13	8.7	67	0.91
Ammonia [1]	1960 - 1972	13	0.047	0.039	0.83	6.4	7.7	0.56
Aniline [1]	1961 - 1972	12	0.027	0.025	0.93	11	12	0.52
Benzene [2]	1953 - 1968	16	0.036	0.027	0.74	8.4	11	0.60
BisphenolA [1]	1959 - 1972	14	0.065	0.027	0.41	4.6	11	0.76
Caprolactam [1]	1962 - 1972	11	0.092	0.050	0.55	3.3	6.0	0.69
CarbonDisulfide [1]	1963 - 1972	10	0.019	0.009	0.47	16	32	0.72
Cyclohexane [1]	1956 - 1972	17	0.060	0.023	0.37	5.0	13	0.77
Ethanolamine [1]	1955 - 1972	18	0.049	0.027	0.53	6.1	11	0.69
EthylAlcohol [1]	1958 - 1972	15	0.031	0.006	0.17	9.8	51	0.89
Ethylene [2]	1954 - 1968	15	0.083	0.016	0.18	3.6	19	0.88
Ethylene2 [1]	1960 - 1972	13	0.058	0.028	0.49	5.2	11	0.71
EthyleneGlycol [1]	1960 - 1972	13	0.041	0.029	0.69	7.3	10	0.62
Formaldehyde [1]	1962 - 1972	11	0.041	0.026	0.63	7.4	12	0.65
HydrofluoricAcid [1]	1962 - 1972	11	0.035	0.001	0.018	8.5	460	0.99
LowDensityPolyethylene [2]	1953 - 1968	16	0.11	0.044	0.38	2.7	6.8	0.77
Magnesium [1]	1954 - 1972	19	0.022	0.003	0.15	13	90	0.90
MaleicAnhydride [1]	1959 - 1972	14	0.055	0.024	0.43	5.4	13	0.74
Methanol [1]	1957 - 1972	16	0.038	0.025	0.68	8.0	12	0.63
NeopreneRubber [1]	1960 - 1972	13	0.033	0.009	0.28	9.1	32	0.82
Paraxylene [2]	1958 - 1968	11	0.10	0.043	0.42	3.0	7.0	0.75
Pentaerythritol [1]	1952 - 1972	21	0.039	0.018	0.45	7.7	17	0.73
Phenol [1]	1959 - 1972	14	0.042	0.035	0.83	7.1	8.5	0.56
PhthalicAnhydride [1]	1955 - 1972	18	0.035	0.031	0.88	8.6	9.7	0.54
PolyesterFiber [1]	1960 - 1972	13	0.12	0.059	0.48	2.4	5.1	0.72
PolyethyleneHD [1]	1958 - 1972	15	0.093	0.042	0.46	3.2	7.1	0.73
PolyethyleneLD [1]	1958 - 1972	15	0.077	0.038	0.50	3.9	7.8	0.71
Polystyrene [2]	1944 - 1968	25	0.086	0.025	0.24	3.5	12	0.84
Polyvinylchloride [2]	1947 - 1968	22	0.073	0.033	0.43	4.1	9.2	0.74
PrimaryAluminum [2]	1930 - 1968	39	0.044	0.011	0.25	6.8	28	0.84
PrimaryMagnesium [2]	1930 - 1968	39	0.075	0.011	0.17	4.0	26	0.89
Sodium [1]	1957 - 1972	16	0.014	0.007	0.47	21	45	0.72
SodiumChlorate [1]	1958 - 1972	15	0.043	0.017	0.40	7.0	17	0.76
Styrene [1]	1958 - 1972	15	0.051	0.030	0.59	5.9	10	0.67
TitaniumSponge [2]	1951 - 1968	18	0.12	0.051	0.38	2.6	5.9	0.77
Urea [1]	1961 - 1972	12	0.065	0.032	0.49	4.6	9.5	0.71
VinylAcetate [1]	1960 - 1972	13	0.055	0.033	0.60	5.5	9.1	0.66
VinylChloride [1]	1962 - 1972	11	0.061	0.039	0.64	5.0	7.7	0.64

Hardware Industry	*time period*	*data points*	g	m	w	*doubling time*	*halving time*	*progress ratio*
DRAM [3]	1972 - 2007	36	0.26	0.19	0.72	1.2	1.6	0.61
HardDiskDrive [4]	1989 - 2007	19	0.28	0.28	1.0	1.1	1.1	0.49
LaserDiode [5]	1983 - 1994	12	0.32	0.14	0.39	0.95	2.2	0.76
Transistor [6]	1969 - 2005	37	0.26	0.21	0.82	1.2	1.4	0.57

Energy Industry	*time period*	*data points*	g	m	w	*doubling time*	*halving time*	*progress ratio*
CCGTElectricity [7]	1987 - 1996	10	0.075	0.009	0.12	4.0	34	0.92
CrudeOil [2]	1947 - 1968	22	0.025	0.004	0.17	12	68	0.89
ElectricPower [2]	1940 - 1968	29	0.046	0.016	0.34	6.5	19	0.79
Ethanol [8]	1981 - 2004	24	0.06	0.023	0.36	5.0	13	0.78
GeothermalElectricity [9] [10]	1980 - 2005	26	0.042	0.022	0.50	7.2	14	0.71
MotorGasoline [2]	1947 - 1968	22	0.028	0.006	0.21	11	48	0.86
OffshoreGasPipeline [11]	1985 - 1995	11	0.11	0.049	0.49	2.7	6.1	0.71
OnshoreGasPipeline [11]	1980 - 1992	13	0.068	0.007	0.11	4.4	45	0.93
Photovoltaics [12]	1976 - 2003	28	0.097	0.028	0.30	3.1	11	0.81
Photovoltaics2 [13]	1977 - 2009	33	0.092	0.045	0.48	3.3	6.7	0.71
WindElectricity [9] [10]	1984 - 2005	22	0.19	0.040	0.18	1.6	7.5	0.88
WindTurbine [14]	1982 - 2000	19	0.12	0.018	0.13	2.5	17	0.91
WindTurbine2 [14]	1988 - 2000	13	0.23	0.017	0.073	1.3	18	0.95

Other Industry	*time period*	*data points*	g	m	w	*doubling time*	*halving time*	*progress ratio*
Beer [2]	1952 - 1968	17	0.077	0.015	0.20	3.9	20	0.87
ElectricRange [2]	1947 - 1967	21	0.029	0.010	0.29	10	31	0.82
FreeStandingGasRange [2]	1947 - 1967	21	0.014	0.009	0.56	21	35	0.68
MonochromeTelevision [2]	1948 - 1968	21	0.074	0.024	0.28	4.1	12	0.82
RefinedCaneSugar [2]	1936 - 1968	33	0.006	0.002	0.32	47	150	0.80

Table 1: Statistics for the datasets used in this study. g is the exponent for the increase in production, m the exponent for the drop in cost, w the exponent for Wright's law, the doubling time refers to the increase in production, the halving time to the decrease in cost, and the progress ratio is 2^{-w}, interpreted as the drop in cost with a doubling of production. All times are in years.

database. Both visualization and free download from the website is available to users. For our analysis we selected the performance curves that had both price and production data for at least 10 years, with no missing values in between. The resulting 62 datasets are products that retained a functional unit equivalence during their (at least decade-long) evolution.

For example, the Transistor dataset represents many different technologies that have been in production during the 37 years in its time period. Transistors have changed dramatically during that period in terms of factors such as speed and power requirements. Nonetheless, one could very crudely say that a transistor as a functional unit is equivalent to any other; hence, we make the crude approximation of comparing the price of such a unit from the sixties to one from the 2000s. Similarly, a bit can be viewed as a functional unit for the DRAM and HardDiskDrive data.

All unit prices are yearly averages after adjusting for inflation. The Transistor, DRAM, HardDiskDrive, and Photovoltaics2 data was converted to real 2005 U.S. dollars using the GDP deflator. The other data sets were published previously, and were converted to real values by their respective authors.

Since originally for each dataset either only yearly production or only total cumulative production was available, we obtained the missing variable from the other one by adding up yearly production or differencing cumulative production, respectively. In order to avoid missing values, this resulted in a shortening of the original time period by one year because for the first year either the previous experience measure was absent (assumed to be zero) or the yearly production was unknown.

2 Exponential increase of production

In this section we present additional evidence for the exponential increase in production, which is one of our new findings in this paper. One way to evaluate the assumption of the constant growth rates g and m and the constant learning rate determined by w is to assess goodness of fit by looking at the distribution of R^2 percentages for the regression lines used for estimating g, m, and w. High R^2 values in Supplementary Figure 1 indicate that in most cases the exponential approximation is accurate for g and m, and the power law fit is good for w.

3 Hindcasting results

We illustrate the hindcasting method using the Transistor dataset. This time series started in the year 1969 and ended in 2005. Thus the first hindcast was made in 1973 based on only five data points, targeting the rest of the time period from 1974 to 2005. The last hindcast was based on the data from 1969 to 2004 having a single target: the year 2005. The resulting projection lines over the actual data points are drawn in Supplementary Figure 2 for the six different functional forms. The most striking feature of Moore's projections is that they consistently underestimated prices for the latter half of the Transistor dataset. On the other hand, Nordhaus's projections strayed from the data in both directions. In contrast, we can see that the other four functional forms demonstrated a more satisfactory prediction performance on this particular dataset, having projection lines near the actual data points and thus avoiding huge deviations (that are measured on the log scale).

Another way to visualize the prediction errors of the hindcasting procedure is to plot them as a surface over two time coordinates: the origin and the target of the hindcasts. Since the target year is always after the origin, the result is a mountain of prediction errors over a triangular area. Figures 3 to 8 use topographical colors to indicate the magnitude of these errors on the log scale

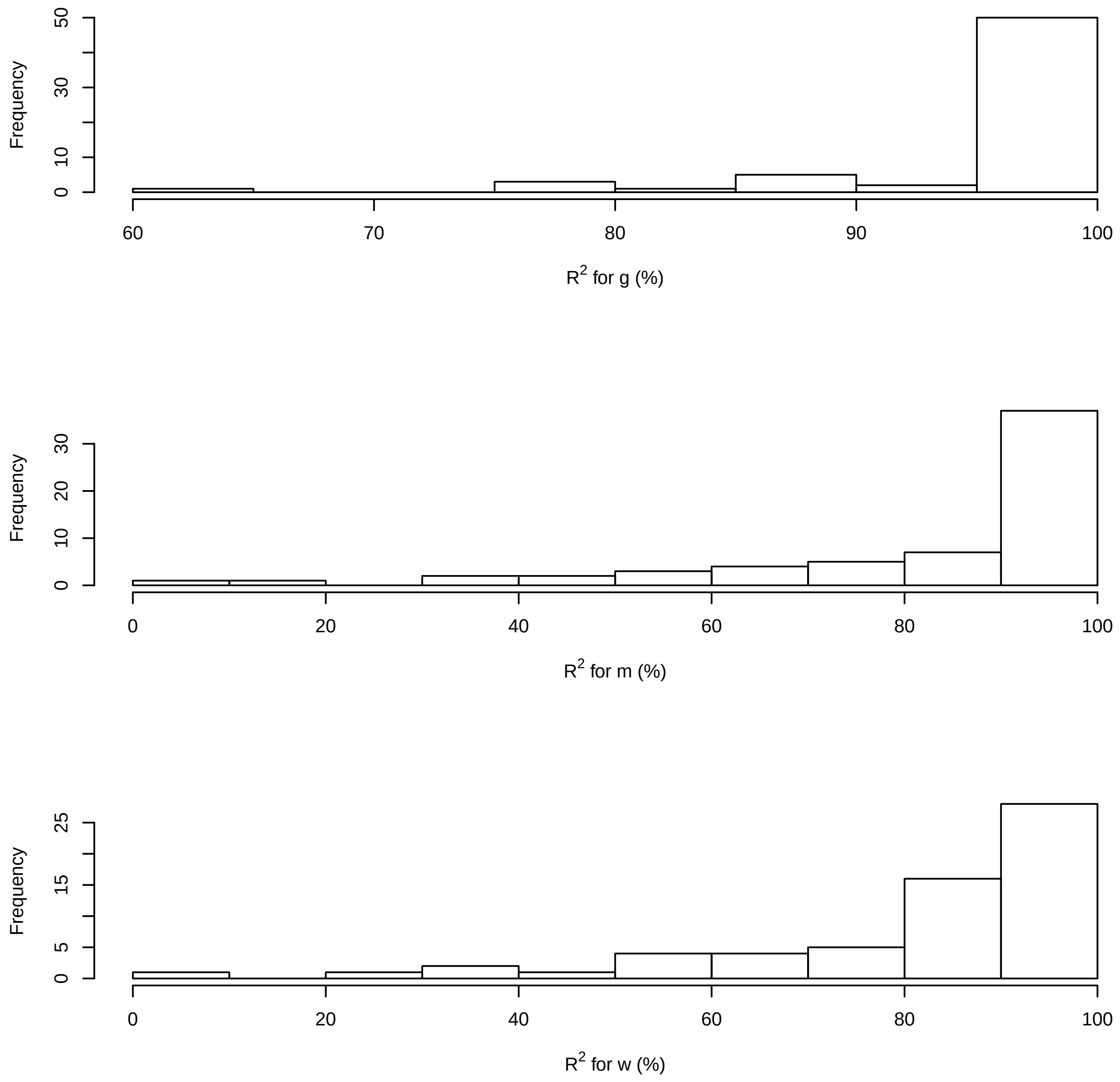

Figure 1: Histograms of R^2 values for fitting g, m, and w for the 62 datasets in percent. The majority of the cases have R^2 values in excess of 90%.

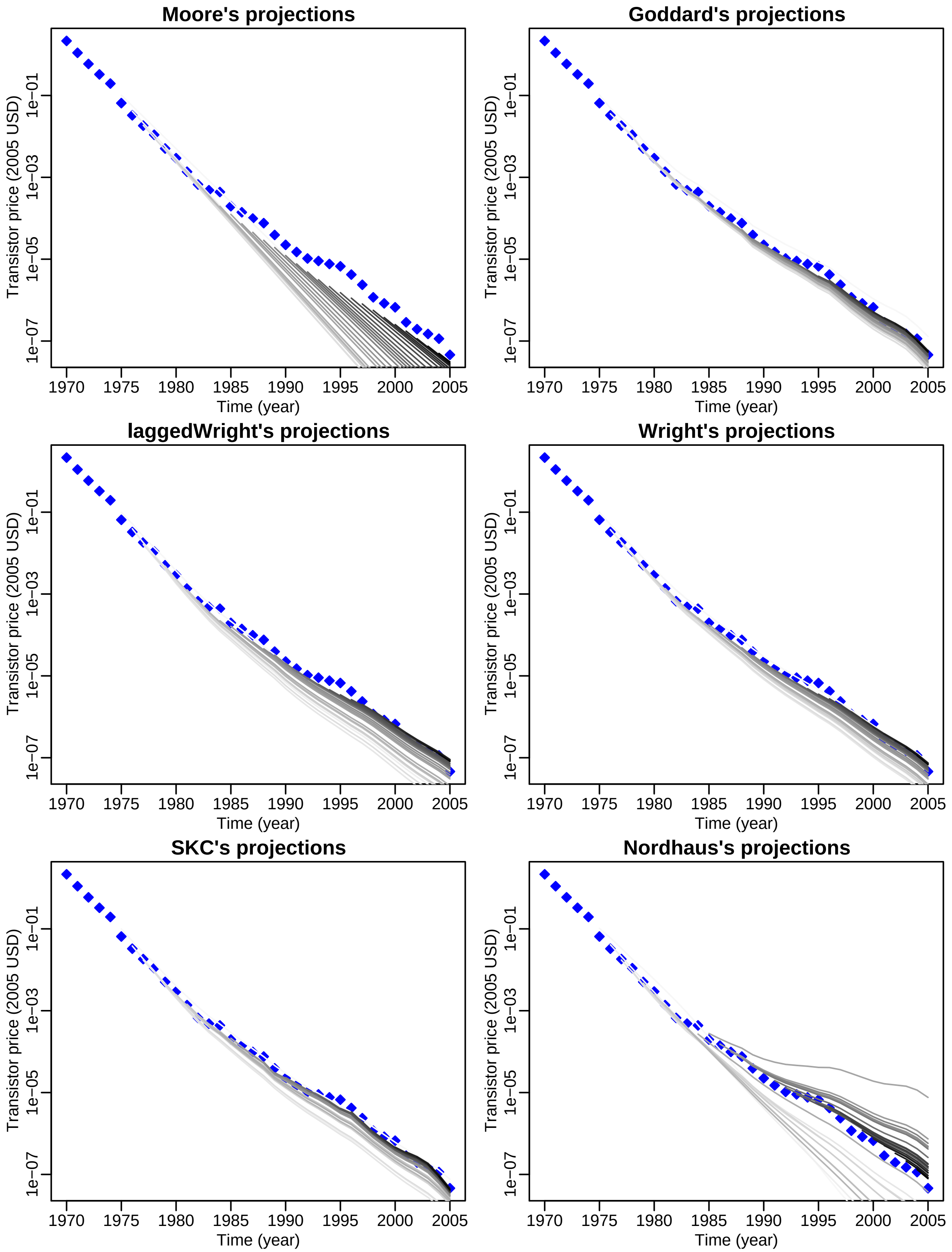

Figure 2: Predictions of six functional forms for the transistor dataset. The hindcasting origin varies from 1973 to 2004 (the target year varies from 1974 to 2005). Forecasts are plotted in gray; the data is shown in blue diamonds. The 1974 forecast is based on five years of data, 1969 - 1973.

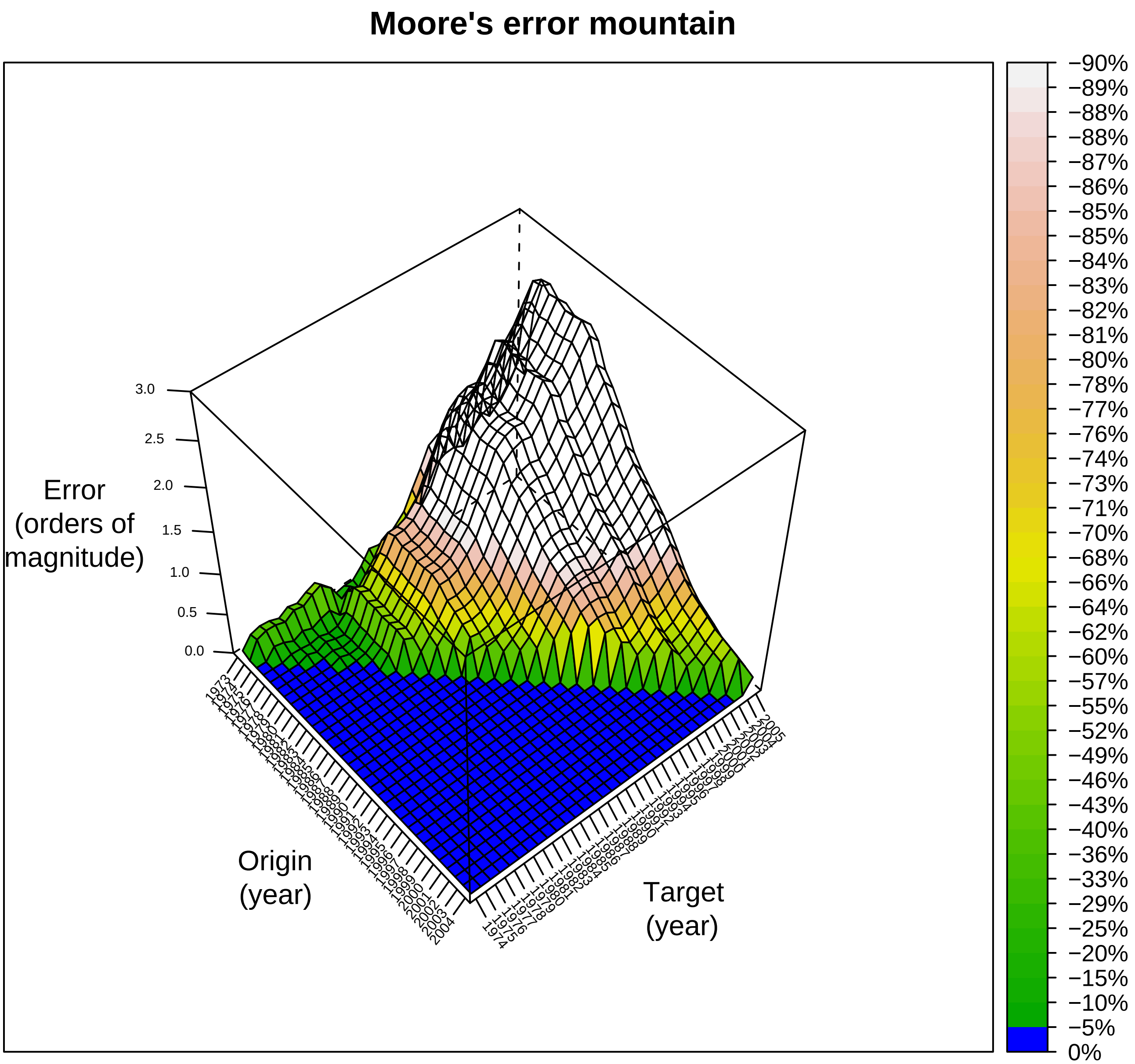

Figure 3: Moore's prediction errors for the Transistor dataset as a function of the hindcasting origin from 1973 to 2004 and the target year from 1974 to 2005.

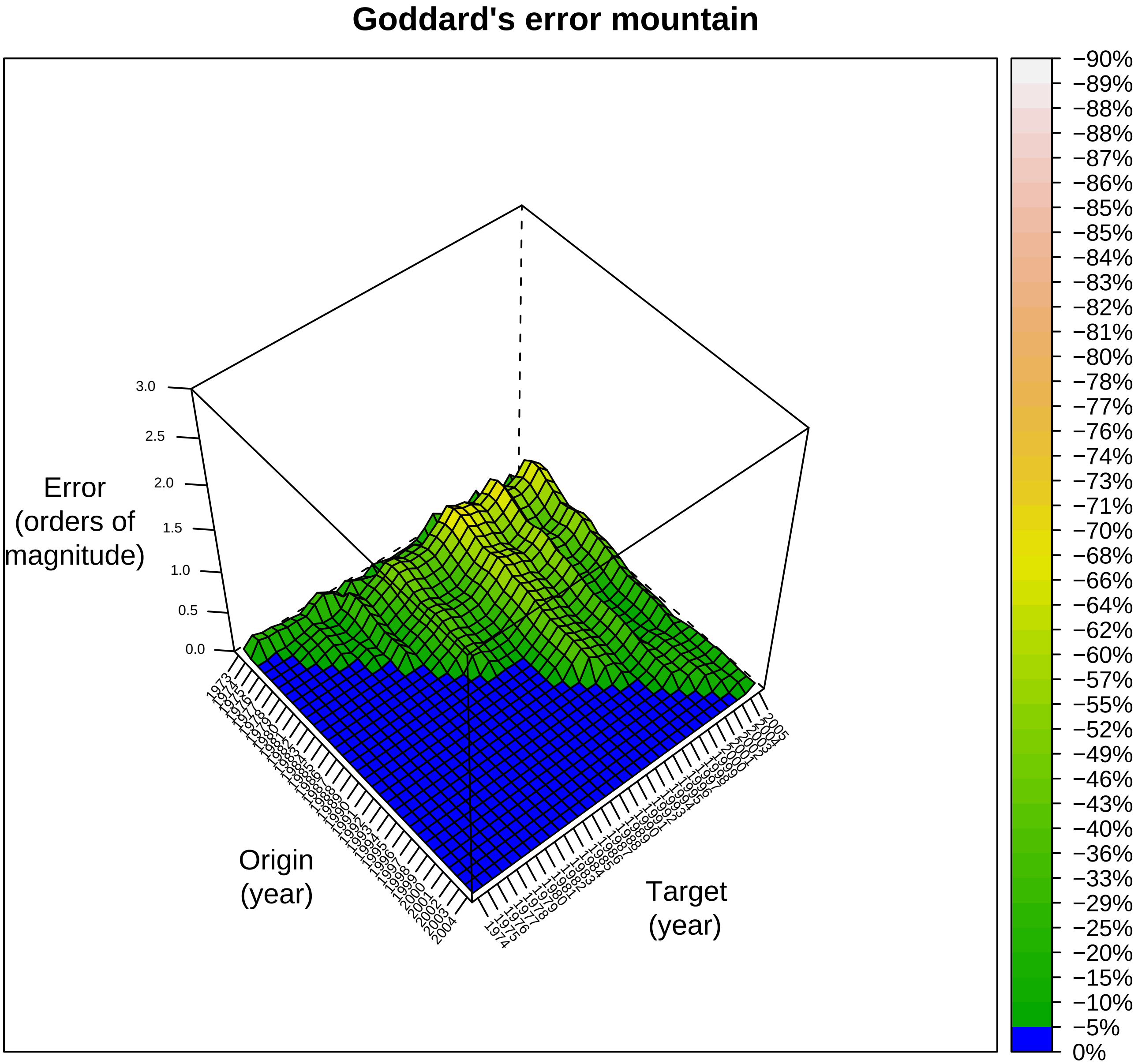

Figure 4: Goddard's prediction errors for the Transistor dataset as a function of the hindcasting origin from 1973 to 2004 and the target year from 1974 to 2005.

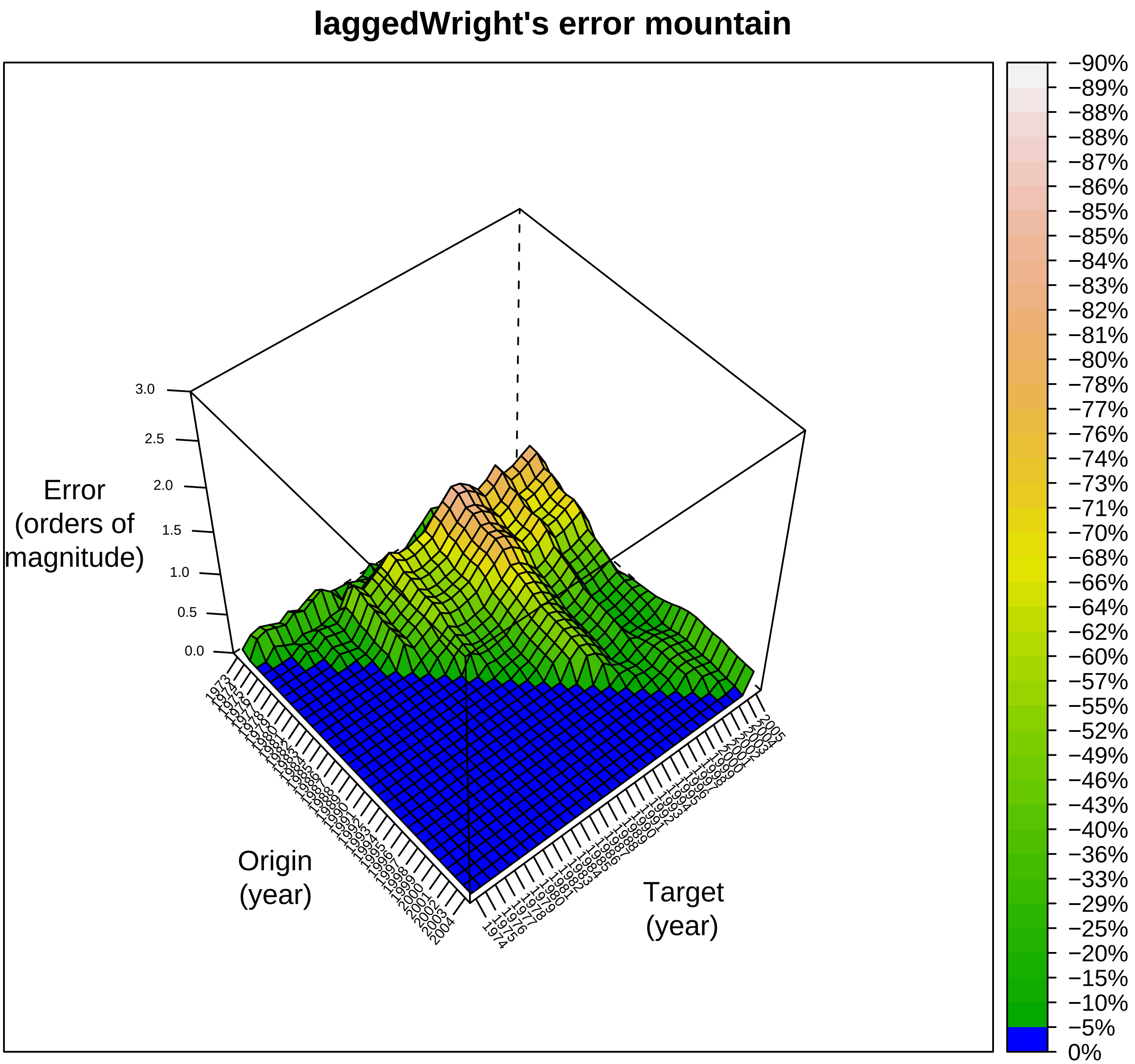

Figure 5: laggedWright's prediction errors for the Transistor dataset as a function of the hindcasting origin from 1973 to 2004 and the target year from 1974 to 2005.

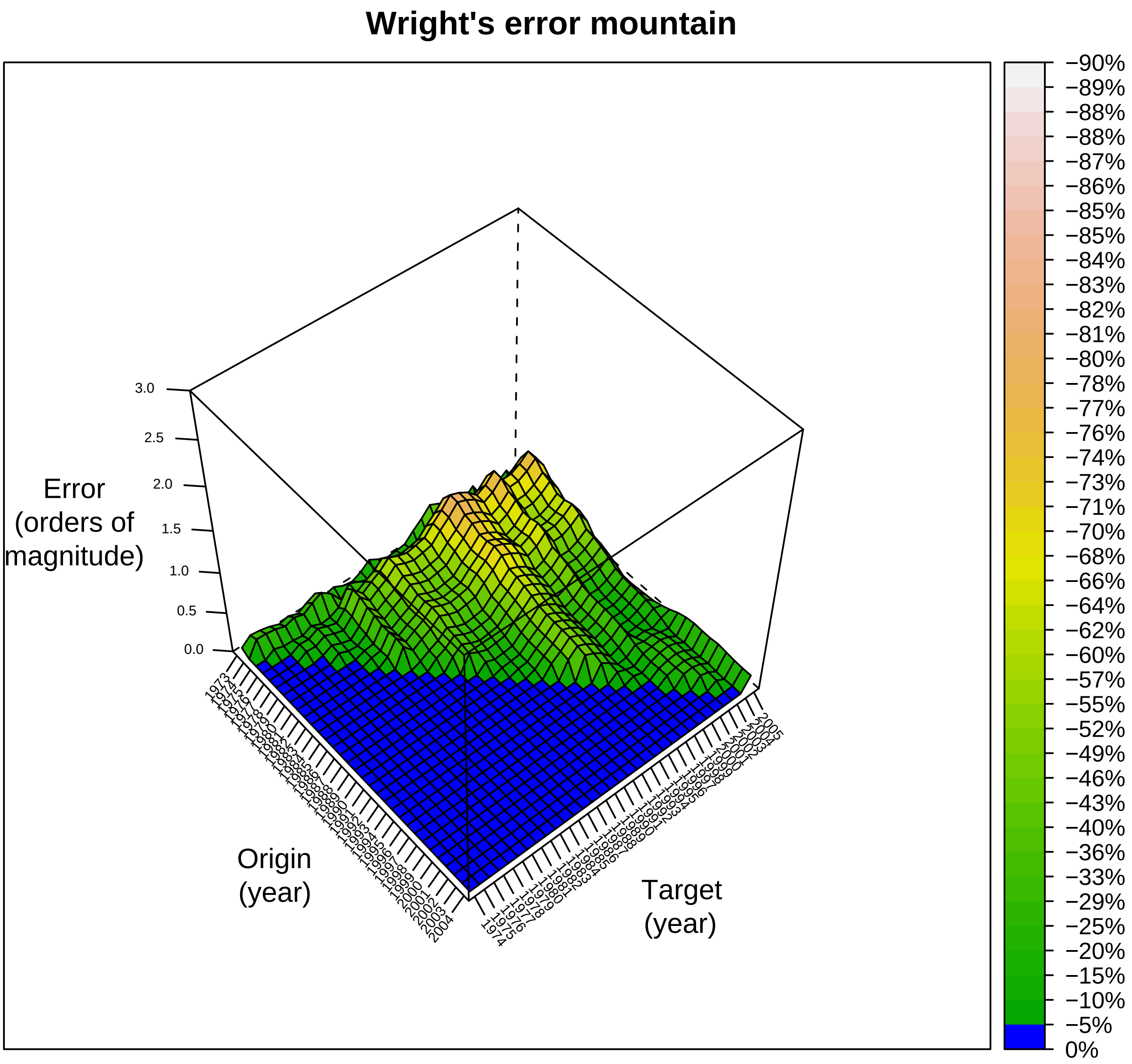

Figure 6: Wright's prediction errors for the Transistor dataset as a function of the hindcasting origin from 1973 to 2004 and the target year from 1974 to 2005.

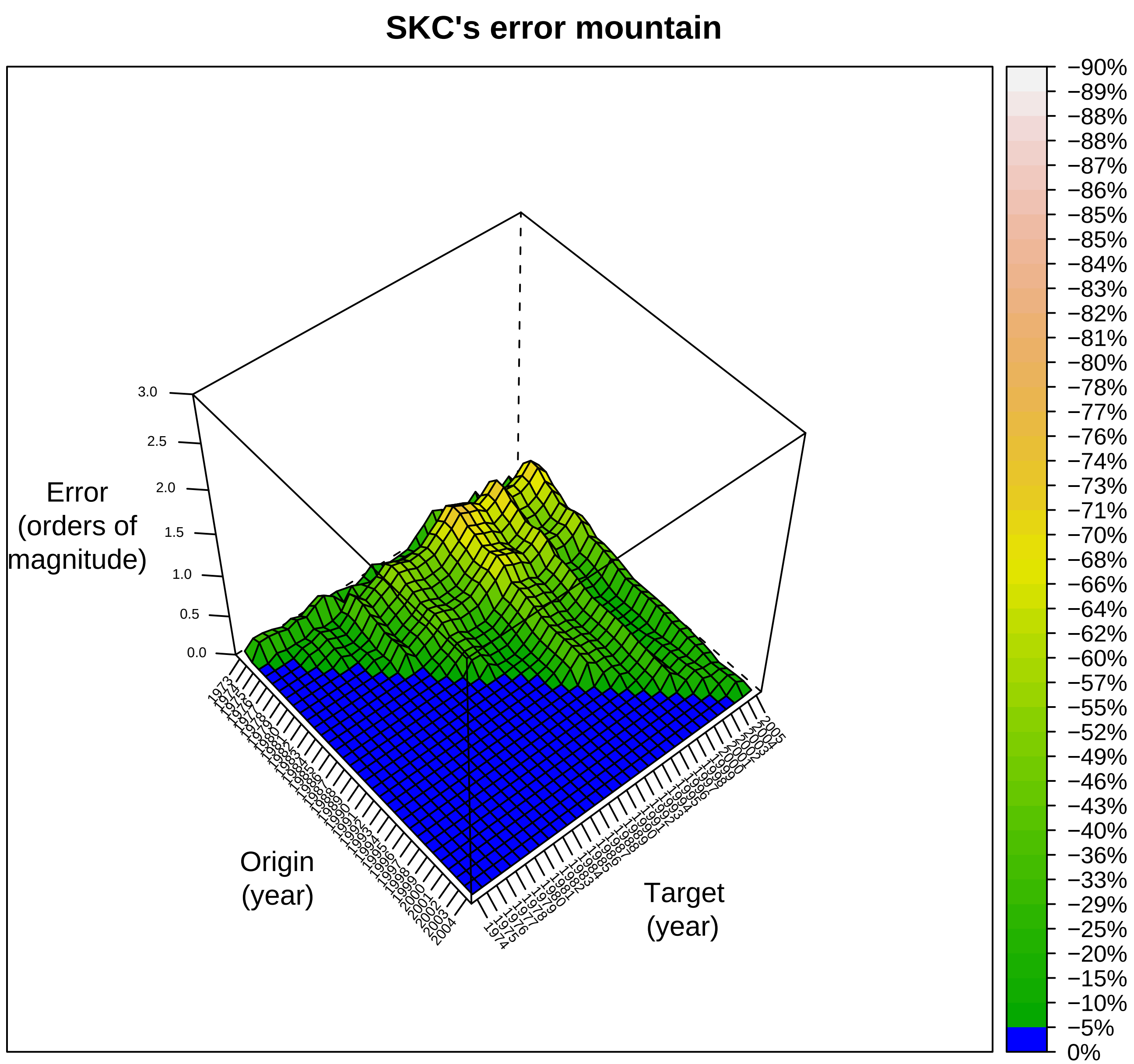

Figure 7: SKC's prediction errors for the Transistor dataset as a function of the hindcasting origin from 1973 to 2004 and the target year from 1974 to 2005.

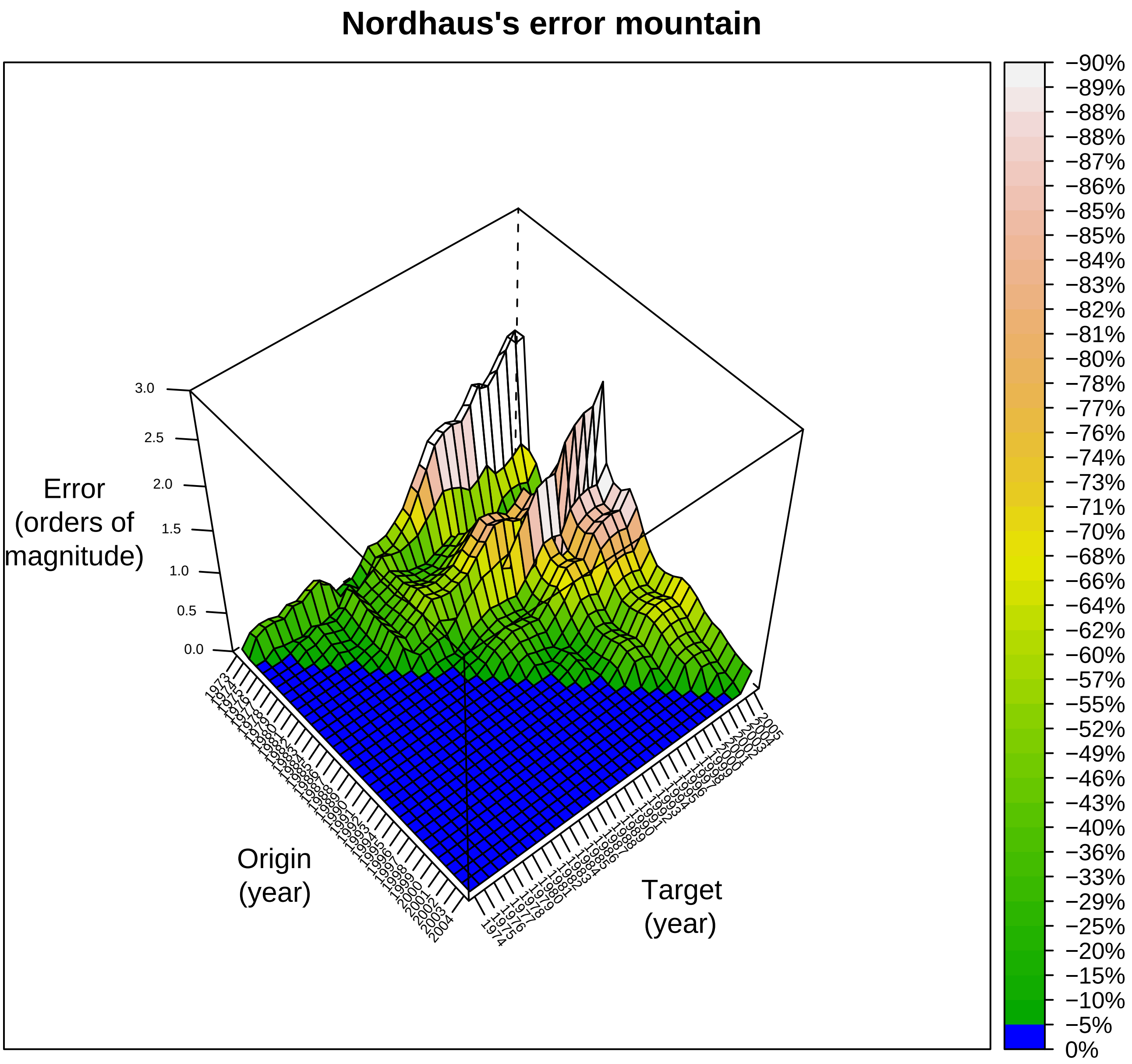

Figure 8: Nordhaus's prediction errors for the Transistor dataset as a function of the hindcasting origin from 1973 to 2004 and the target year from 1974 to 2005.

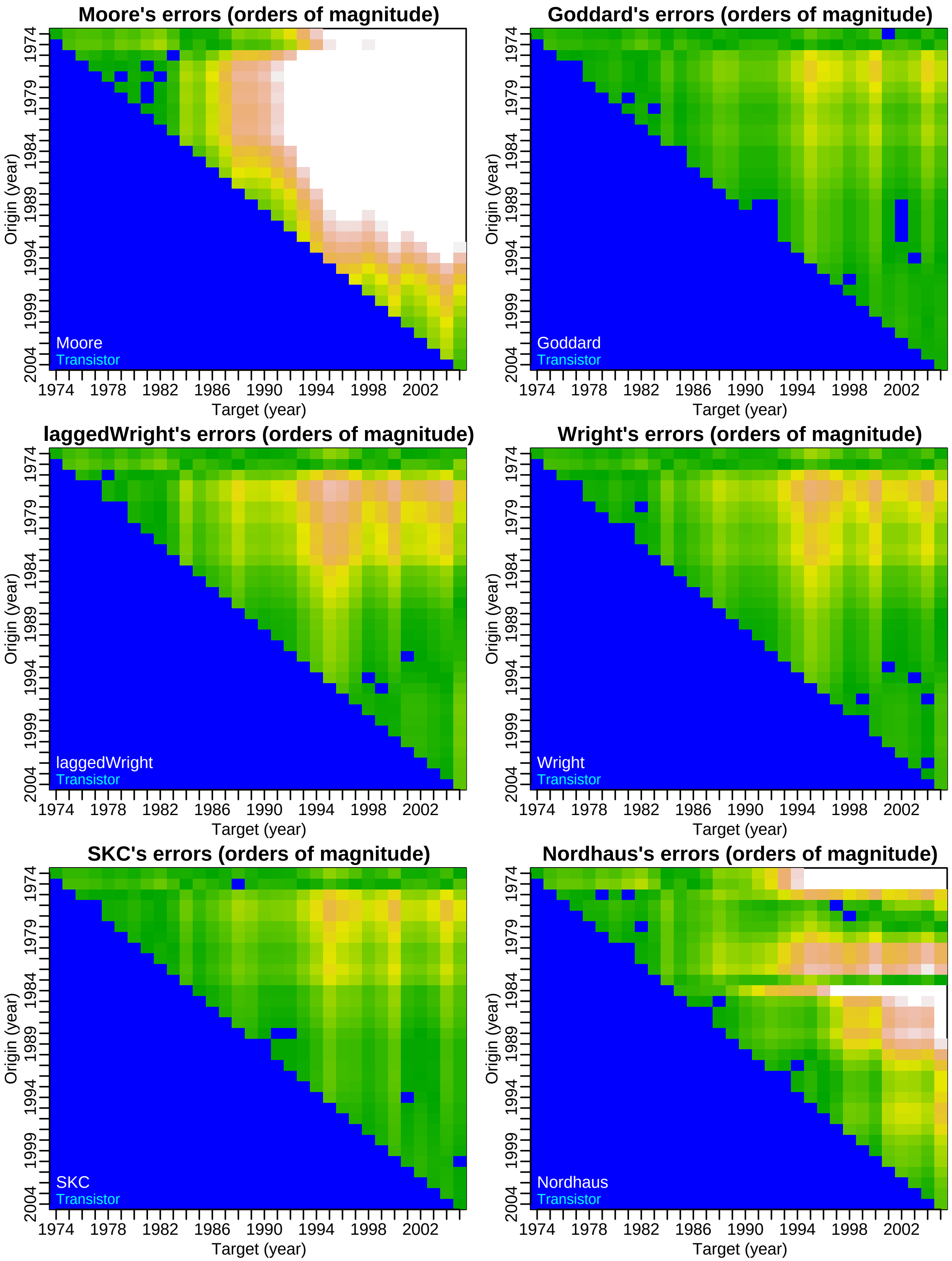

Figure 9: Summary of the prediction errors of six functional forms for the Transistor dataset as a function of the hindcasting origin from 1973 to 2004 and the target year from 1974 to 2005.

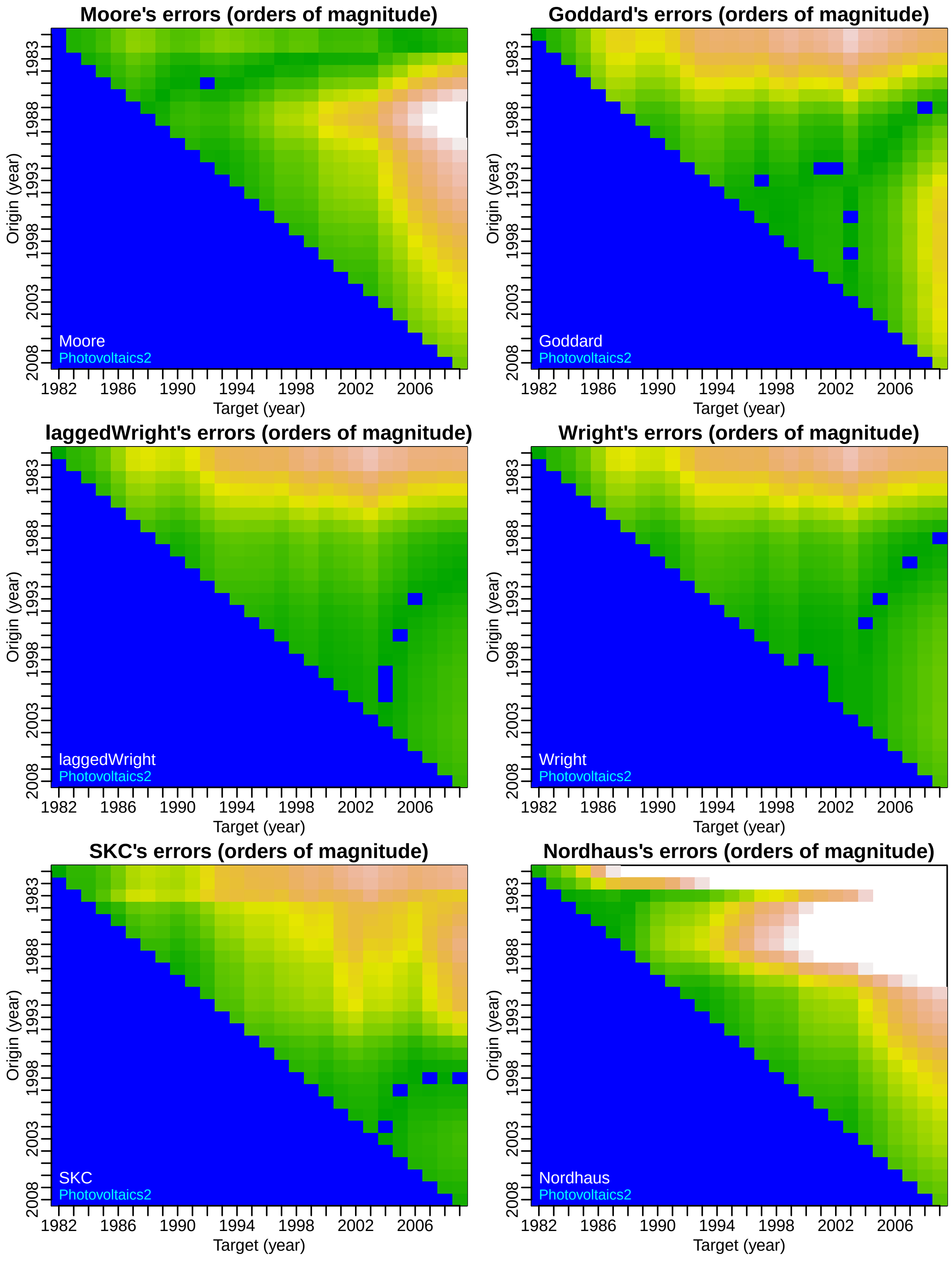

Figure 10: Summary of the prediction errors of six functional forms for the Photovoltaics2 dataset as a function of the hindcasting origin from 1981 to 2008 and the target year from 1982 to 2009.

(base 10). Note that the coloring scheme only extends from the sea level in blue (meaning no or negligible error between -6% and +6%), up to the snow line at 1.0 (above which the rest of the mountain is left uncolored). This is where the absolute error on the log scale reaches 1.0, meaning that the predicted price is either 10 times greater than the actual (+900% hindcasting error) or 10 times less (−90% error). To illustrate how the errors on the log scale translate to percentages, the color key on the right of these three-dimensional plots indicate the negative error range on the original scale from 0% to −90%. (However, we should keep in mind that because the logarithm of the absolute difference is taken, a 1.0 on the log scale can mean either +900% or −90%).

The functional form inspired by Moore does not predict transistors very well. This is ironic since transistors are the technology that Moore's Law was originally formulated to describe. In fact, among all the 62 performance curves, Moore's functional form was the least accurate for the Transistor dataset. We can see that a large portion of the error mountain in Figure 3 rises not only above the 1.0 mark at the snow line, but also exceeds the 2.0 mark, i.e. hindcasting that the price would be less than one-hundredth of what it actually turned out to be. The summit is at 2.67, meaning that in this case the actual price is underestimated by a factor of 467.

For a side-by-side comparison of all the hindcasts on the Transistor dataset, Supplementary Figure 9 is a bird's-eye view from the top of how the six competing forms fared against one another. Supplementary Figure 10 is a similar plot for the Photovoltaics2 dataset. Supplementary Figures 11 to 16 are top view supergraphics for the error mountains generated by the six functional forms for the other 60 datasets.

4 Error model

Visualizing the error mountains is a quick and intuitive way to screen out inadequate functional forms like Nordhaus that show erratic behavior. This was especially easy to do with multiple-variable forms (with or without interaction terms) that were prone to overfitting, which is manifested by the fact they gave good in-sample fits but generated large and inconsistent errors when trying to predict out-of-sample. Hence, Nordhaus and all the other multiple-variable forms that failed to generate a relatively consistent prediction error surface (without huge jumps) were not included in the subsequent formal analysis. The statistical comparison we made here in constructing our error model is only between the “finalists".

After ruling out all multiple-variable forms but SKC's by visual inspections of the error mountains, we are left with five candidates competing for the hindcasting champion title: Moore, Goddard, laggedWright, Wright, and SKC. For example, does Moore's comparably weaker performance on the Transistor dataset make it an inferior functional form? How do the others compare? Is any one of them significantly better than any of the others?

There are no obvious ways to answer these questions, but one way is to build a suitable statistical model for the errors generated by the remaining five functional forms, based on the data displayed for those functional forms in Supplementary Figures 9 to 16. The statistical model employed here is an extended linear mixed-effects model, fitted by maximum likelihood, using the *lme* function in the *nlme* package in R. The mixed-effects designation here refers to the presence of both fixed and random effects (as explained later in this section). The basic linear mixed-effects model needed extension because the hindcasting data was both heteroscedastic (with unequal variances) and correlated (not independent).

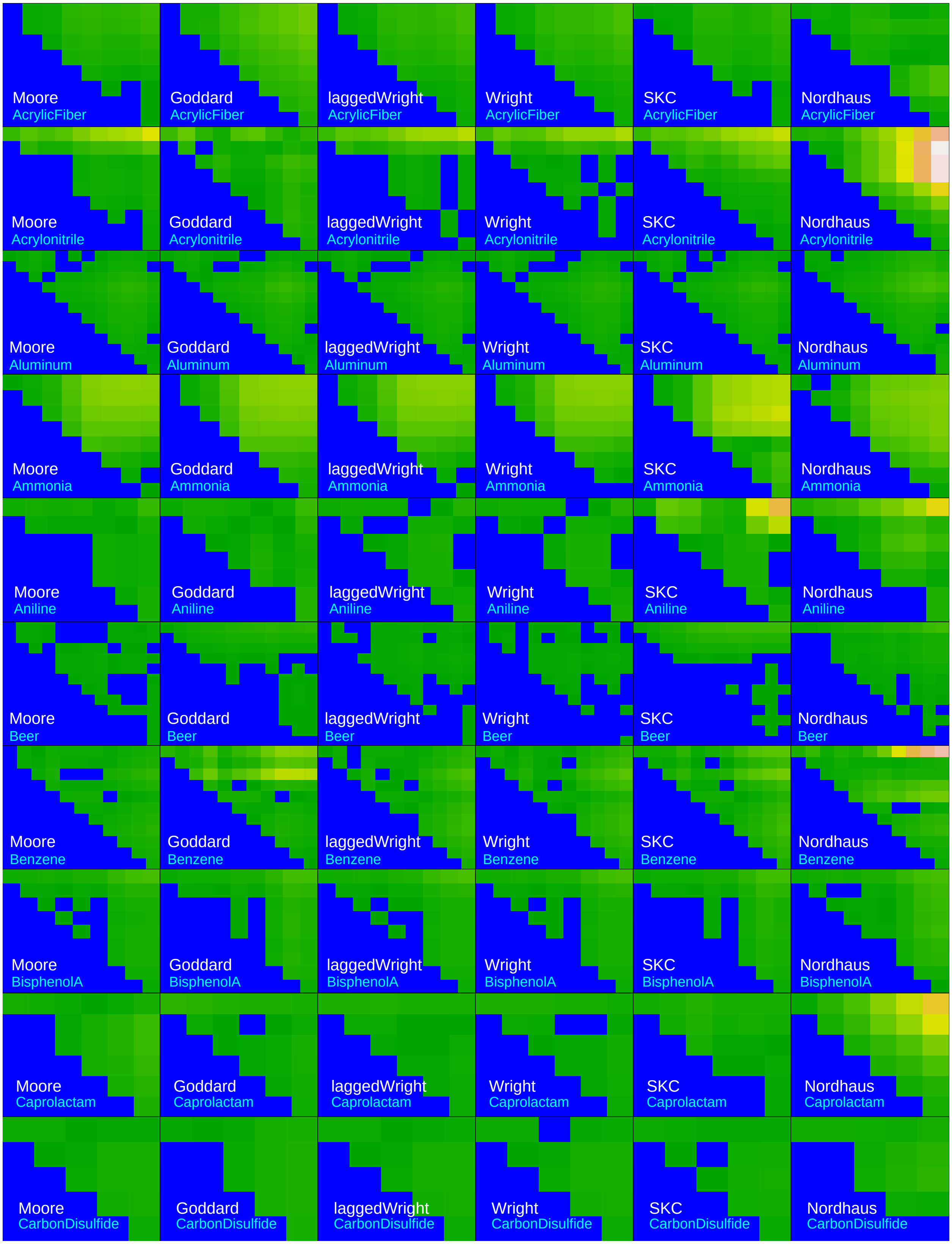

Figure 11: Summary of the prediction errors generated by six functional forms as a function of the hindcasting origin (vertical axis) and the target year (horizontal axis).

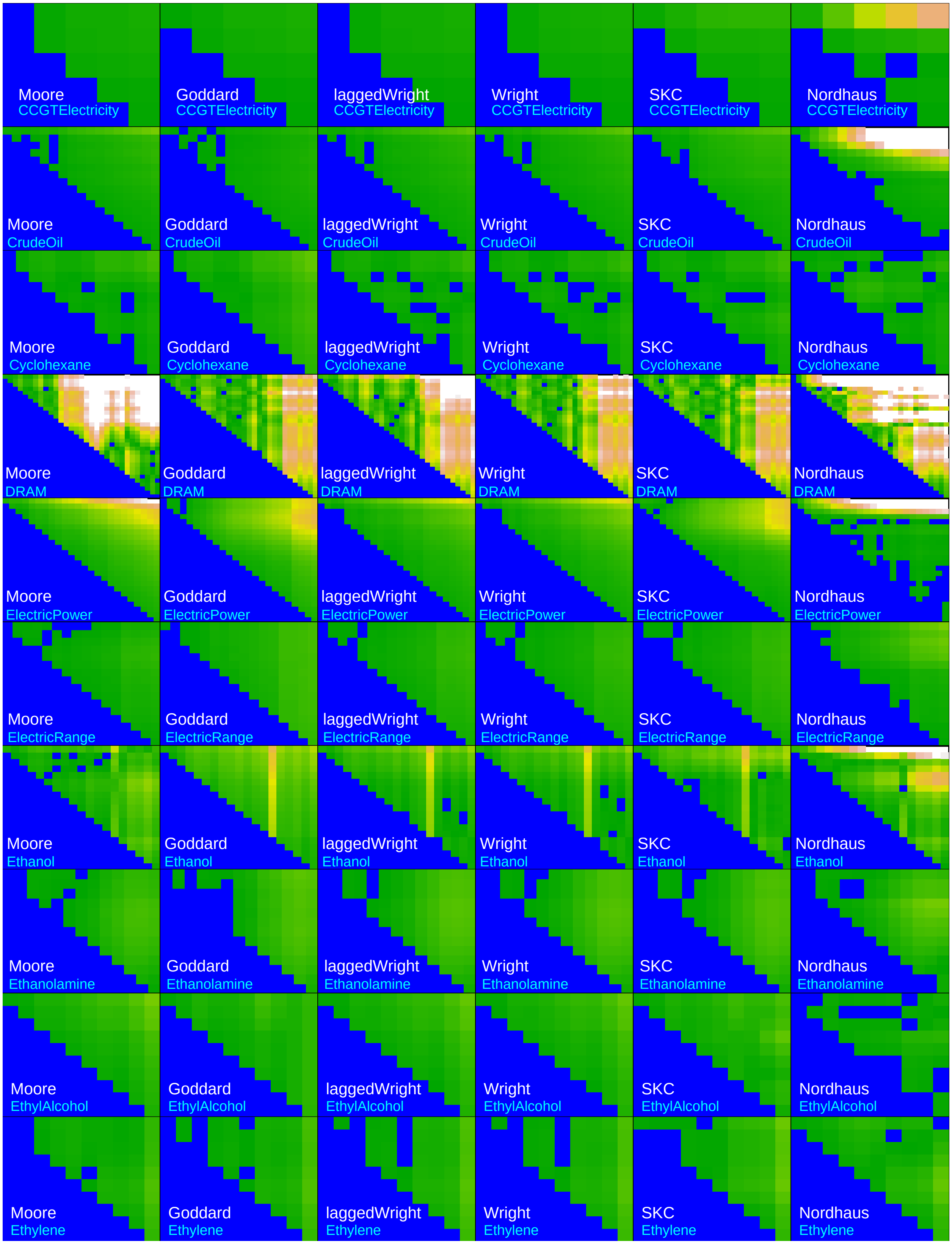

Figure 12: Summary of the prediction errors generated by six functional forms as a function of the hindcasting origin (vertical axis) and the target year (horizontal axis).

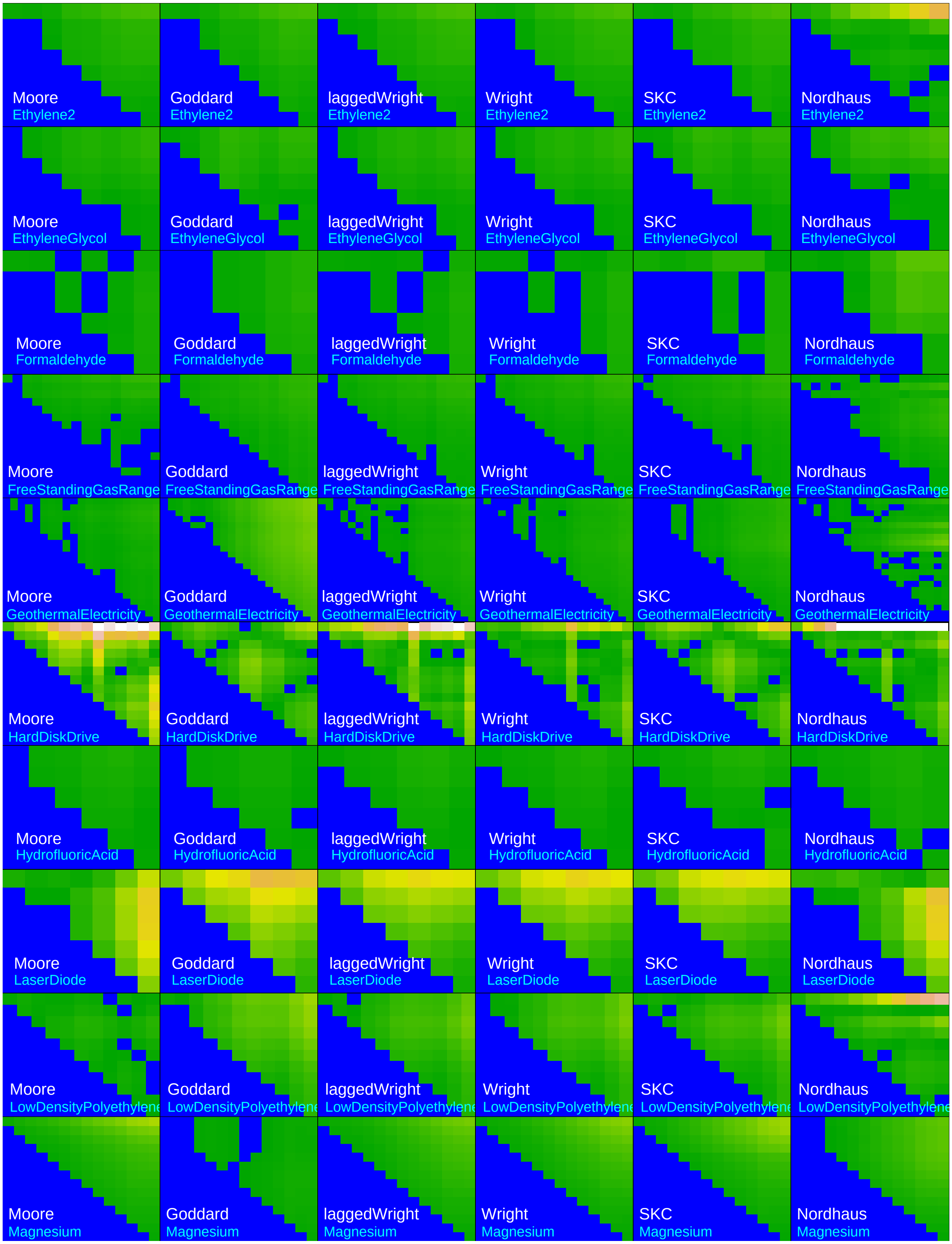

Figure 13: Summary of the prediction errors generated by six functional forms as a function of the hindcasting origin (vertical axis) and the target year (horizontal axis).

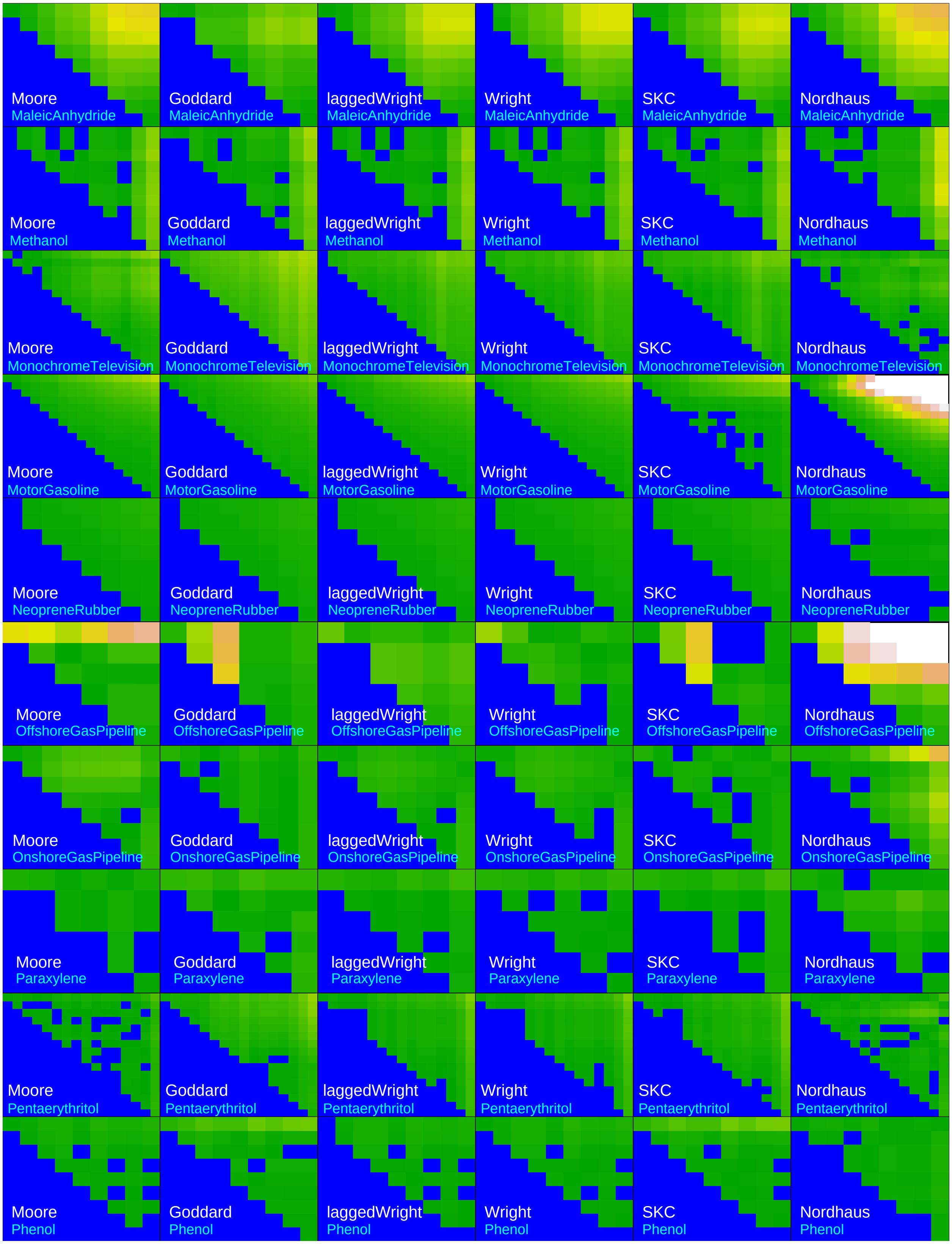

Figure 14: Summary of the prediction errors generated by six functional forms as a function of the hindcasting origin (vertical axis) and the target year (horizontal axis).

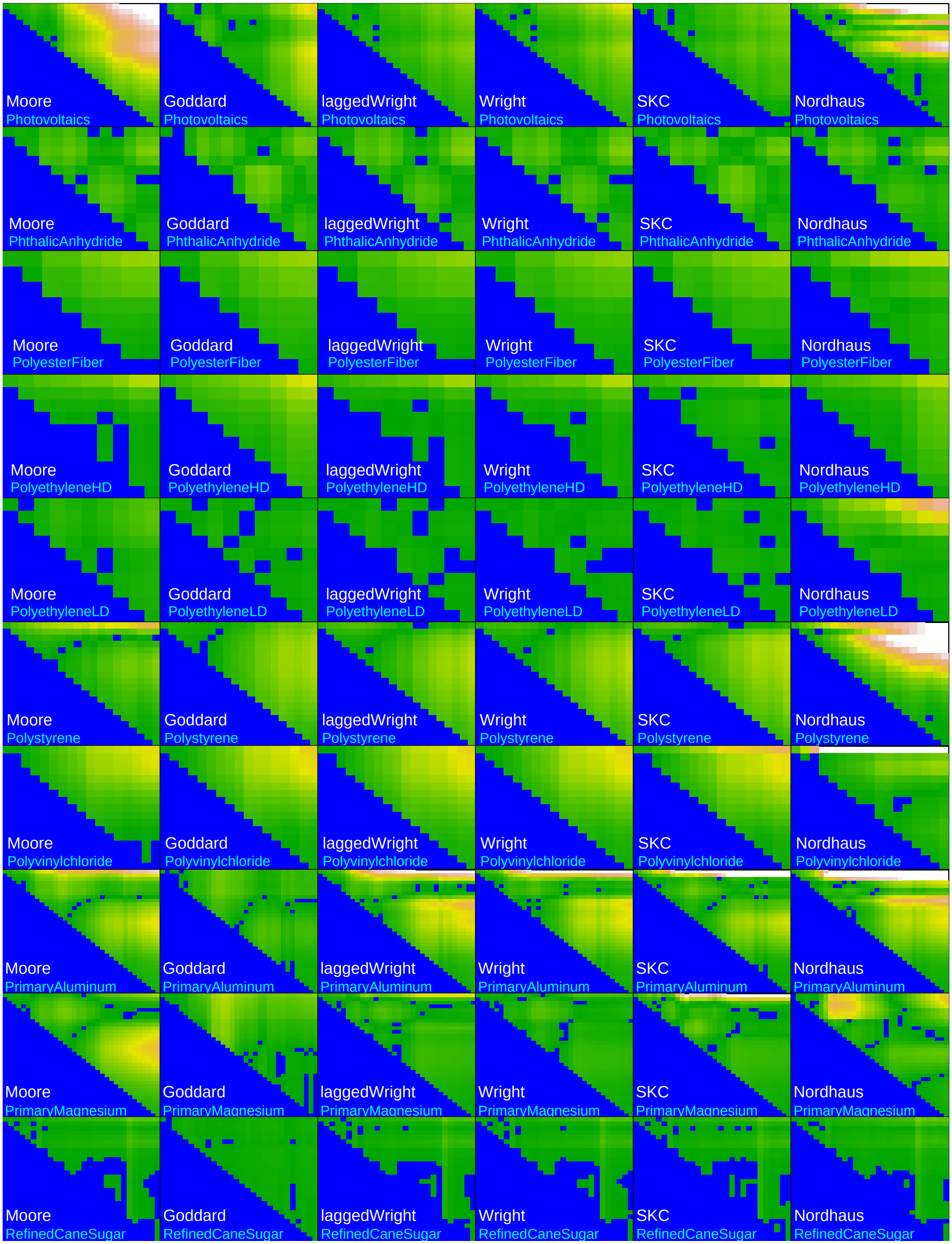

Figure 15: Summary of the prediction errors generated by six functional forms as a function of the hindcasting origin (vertical axis) and the target year (horizontal axis).

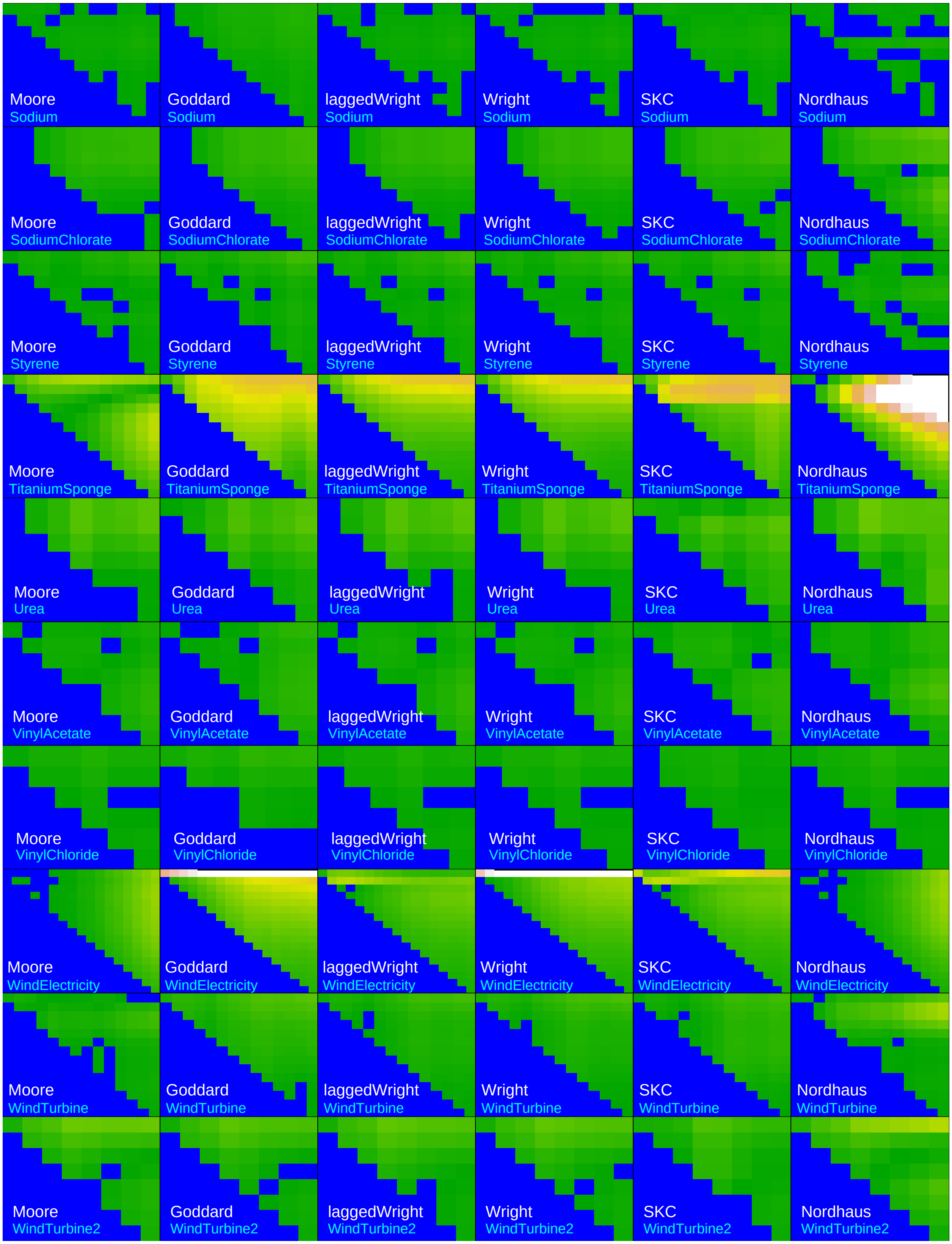

Figure 16: Summary of the prediction errors generated by six functional forms as a function of the hindcasting origin (vertical axis) and the target year (horizontal axis).

4.1 Choosing the response

When trying to build a model that captures the essence of a large dataset, one is faced with many theoretical and practical challenges, involving several subjective decisions, such as the various tradeoffs between simplicity and goodness of fit. One of the most important early choices is what to model in the first place. We started out by trying to model the prediction errors directly (i.e. the height of the error mountains), but that did not lead to acceptable diagnostics of the resulting fits.

Next we experimented with transforming these prediction errors in order to obtain a response that will allow a better fit. We searched the family of power transformations, which is known for its flexibility to accommodate a wide range of variance structures for the purposes of linear modeling. Exponents in the neighborhood of 0.5 provided the best fits in terms of regression diagnostics, so we fixed it at 0.5. This meant applying a square root transformation to the original errors.

Formally, the response r_{fdij} used in this model is the root-transformed absolute hindcasting error on the log scale (base 10) of the hindcast made in year i for year j using the functional form f for dataset d. In other words, this is the square root of the height of the error mountain of f for d at the time coordinates i and j:

$$r_{fdij} = \left|\log y_j^{(d)} - \log \hat{y}_j^{(f,d,i)}\right|^{0.5}, \tag{1}$$

where $y_j^{(d)}$ is the actual price in the dataset d in year j and $\hat{y}_j^{(f,d,i)}$ is the price estimated by the functional form f for year j, using all data in d available up to and including year i, where $i < j$.

4.2 Modeling the response

The main advantage of choosing the response given by equation (1) is that it can be modeled in a parsimonious manner as a linear function of the hindcasting horizon $=$ target $-$ origin $= j - i$. The effect of each functional form f can be characterized by two numbers: an intercept α_f and a slope parameter β_f that specifies this linear relationship. But the individual curves themselves can have large effects on the response and we need to take that into account, too. So, instead of modeling only the average effect of the functional form f by a linear function of the hindcasting horizon $(j-i)$ with the linear relationship $\alpha_f + \beta_f(j-i)$, we model the joint effect of the functional form f and the performance curve data d with the adjusted linear trend $(\alpha_f + a_d) + (\beta_f + b_d)(j-i)$, where the a_d and b_d quantities are additive adjustments to the average intercept and slope parameters α_f and β_f, respectively, to take into account the peculiarities of the dataset d.

In order to avoid adding 62 a_d parameters plus 62 b_d parameters, we treated the $\begin{pmatrix} a_d \\ b_d \end{pmatrix}$ pair as a two-dimensional random vector having a bivariate normal distribution with mean $\begin{pmatrix} 0 \\ 0 \end{pmatrix}$ and variance-covariance matrix $\begin{pmatrix} \psi_a^2 & \psi_{ab} \\ \psi_{ab} & \psi_b^2 \end{pmatrix}$. This way we can parameterize these adjustments as random deviations from the average $\begin{pmatrix} \alpha_f \\ \beta_f \end{pmatrix}$ at a cost of only 3 additional parameters instead of $2 \times 62 = 124$, resulting in a parameterization that is not only much more parsimonious but also makes maximum likelihood estimation possible by keeping the number of parameters in check.

In statistical terminology, we can say that the effects of the 62 performance curves are random (as opposed to the fixed effects of the five functional forms). The interpretation is that we view

each curve as a random draw from a hypothetical population of performance curves. Of course, the underlying assumption here is that it makes sense to talk about such an ensemble of performance curves that have enough characteristics in common to make this notion meaningful.

4.3 Statistical model

Now we are ready to define the extended linear mixed-effects model by the following equation:

$$r_{fdij} \; = \; (\alpha_f + a_d) + (\beta_f + b_d)(j - i) + \varepsilon_{fdij}, \tag{2}$$

where the response is modeled as the sum of a trend term (that is a linear function of the hindcasting horizon $(j - i)$ as before) plus an ε_{fdij} random field term to take into account the deviations from the trend. This is assumed to be a Gaussian stochastic process independent of the $\begin{pmatrix} a_d \\ b_d \end{pmatrix}$ random vector, having mean 0, and given a_d and b_d, having variance equal to a positive σ^2 times the fitted values:

$$\mathrm{Var}\,(\varepsilon_{fdij}|\, a_d, b_d) \; = \; \sigma^2\, \mathrm{E}\,(r_{fdij}|\, a_d, b_d) \tag{3}$$

and an exponential correlation structure within each mountain that is a function of the differences of the two time coordinates with a positive range parameter ρ and another small positive nugget parameter η quantifying the extent of these correlations:

$$\mathrm{Corr}(\varepsilon_{fdij},\, \varepsilon_{f'd'i'j'}) \; = \; \delta_{ff'}\delta_{dd'}(1 - \eta)\exp\{-(|i - i'| + |j - j'|)/\rho\}, \tag{4}$$

where the two Kronecker δ functions ensure that each mountain surface is treated as a separate entity.

Equations (3) and (4) were chosen to deal with the fact that variances tend to increase with altitude on the error mountains and that there are serial correlations along the time coordinates i (hindcasting origin) and j (hindcasting target). The heteroscedasticity (increasing variance with increasing elevation) problem is handled by the variance function (3) and the time dependence is taken care of by the correlation function (4). Based on the likelihood, this exponential correlation function provided the best fit. Note that instead of the usual Euclidean distance (root sum of squares of differences), here the so-called “Manhattan” measure was used (the sum of the absolute differences), because it provided a much better fit in terms of the likelihood.

4.4 Intercept and slope parameter estimates

The maximum likelihood estimates for the five intercept and five slope parameters are listed in Tables 2 and 4, respectively. It is evident that all five functional forms perform similarly in terms of hindcasting accuracy, because most of these estimates are not significantly different from one another. The corresponding approximate p-values for all pairwise comparisons are listed in Tables 3 and 5. The highest intercept estimate for Goddard means that it does a relatively poor job of forecasting at short times, whereas the higher slope estimates for Moore and SKC mean that they are not as good at long times. Otherwise the models are roughly equivalent.

5 Extrapolation method

For the purposes of this paper we have chosen to fit the model to all the past data available at time i and use the resulting parameter estimates to make the forecasts. Thus the forecast corresponds to

Intercept	*estimate*
α_{SKC}	0.164
α_{Moore}	0.168
α_{Wright}	0.170
$\alpha_{\text{laggedWright}}$	0.172
α_{Goddard}	0.195

Table 2: Intercept estimates.

Intercept difference	*p-value*
$\alpha_{\text{Moore}} - \alpha_{\text{SKC}}$	0.555
$\alpha_{\text{laggedWright}} - \alpha_{\text{SKC}}$	0.224
$\alpha_{\text{laggedWright}} - \alpha_{\text{Moore}}$	0.531
$\alpha_{\text{Wright}} - \alpha_{\text{SKC}}$	0.389
$\alpha_{\text{Wright}} - \alpha_{\text{Moore}}$	0.786
$\alpha_{\text{Wright}} - \alpha_{\text{laggedWright}}$	0.722
$\alpha_{\text{Goddard}} - \alpha_{\text{SKC}}$	0.000
$\alpha_{\text{Goddard}} - \alpha_{\text{Moore}}$	0.001
$\alpha_{\text{Goddard}} - \alpha_{\text{laggedWright}}$	0.005
$\alpha_{\text{Goddard}} - \alpha_{\text{Wright}}$	0.002

Table 3: Testing whether the pairwise intercept differences are significantly different from zero.

the trend line for the entire data set, and points in the distant past get just as much weight as points in the present. We have chosen this method because it is the “vanilla" model, and probably the most widely used to apply these laws. This method is also well-suited to compare standard hypotheses, which is the main objective of the paper. We do not argue that this is the best possible forecasting method; developing more accurate forecasting methods will be a topic of future research. In the meantime we want to provide more background information on some of the anomalies commented on in the text, and in particular figure 5 and footnote 7.

In figure 5 there is an immediate drop in the one-year forecast relative to the last observed price. This is a direct consequence of the use of the vanilla method, which is the ideal model if the data are generated by independent fluctuations around a deterministic trend. To illustrate this with Moore’s law, suppose the true random process generating the data is of the form:

$$\log y_t = at + n(t). \tag{5}$$

This is the most straightforward interpretation of Moore’s law. If the noise terms $n(t)$ are uncorrelated in time, then the method we have used to make forecasts here is ideal. But if the noise terms are correlated in time this is no longer the case. Suppose, for example, that the process is better described by a random walk with drift, of the form

$$\log y_{t+1} = \log y_t - \mu + n(t), \tag{6}$$

where μ is a drift term, and where the noise fluctuations $n(t)$ are uncorrelated in time. In this case the best forecast for $\log y_{t-1}$ is $\log y_t - \mu$. There are many intermediate possibilities, for example if $\log y_t$ is a long-memory process.

We find clear evidence for memory in the noise terms. Taking into account correlations in the noise terms produces better forecasts for short time horizons. For longer time horizons, greater than 3 - 5 years, they are roughly equivalent.

Slope	*estimate*
β_{Goddard}	0.02396
β_{Wright}	0.02397
$\beta_{\text{laggedWright}}$	0.02438
β_{Moore}	0.02706
β_{SKC}	0.02848

Table 4: Slope estimates.

Slope difference	*p-value*
$\beta_{\text{Moore}} - \beta_{\text{SKC}}$	0.394
$\beta_{\text{laggedWright}} - \beta_{\text{SKC}}$	0.011
$\beta_{\text{laggedWright}} - \beta_{\text{Moore}}$	0.093
$\beta_{\text{Wright}} - \beta_{\text{SKC}}$	0.005
$\beta_{\text{Wright}} - \beta_{\text{Moore}}$	0.050
$\beta_{\text{Wright}} - \beta_{\text{laggedWright}}$	0.788
$\beta_{\text{Goddard}} - \beta_{\text{SKC}}$	0.007
$\beta_{\text{Goddard}} - \beta_{\text{Moore}}$	0.059
$\beta_{\text{Goddard}} - \beta_{\text{laggedWright}}$	0.788
$\beta_{\text{Goddard}} - \beta_{\text{Wright}}$	0.991

Table 5: Testing whether the pairwise slope differences are significantly different from zero.

The use of the vanilla model leads to some peculiar results. For example, we find that short term forecasts get worse as we add more historical data — in other words, recent data is more useful than data in the far past. This is not surprising if the true dynamics are closer to Eq. 6 than to Eq. 5 — more data systematically means that the most recent point will show larger deviations from the trend line. However, continually adjusting trend lines to take into account the most recent data compromised the goodness of fit of our error model by generating excessive noise (discontinuities in the error mountains). In other words, improving the short-term forecasts would have compromised our ability to compare standard hypotheses (functional forms commonly used to forecast technological improvement).

In Figure 5, we are using one of the longest time series in the data set, and the forecast is based on the entire series. Thus the errors for a time horizon of one are large compared with the typical series in the data set. This is not a problem at longer time horizons. The error estimates after the first five years become more trustworthy.

In future we may work on constructing a “best model". In this paper, our goal is to place the problem of forecasting technological change using past performance in a solid statistical context, and to quantify the quality of the forecasts.

References

[1] Lieberman MB (1984) The learning curve and pricing in the chemical processing industries. The RAND Journal of Economics 15: 213–228.

[2] Group BC (1972) Perspectives on Experience. One Boston Place, Boston, Massachusetts 02106: The Boston Consulting Group, Inc., 3 edition.

[3] Cullen S (2008). Personal communication.

[4] Coughlin T (2008). Personal communication.

[5] Lipman TE, Sperling D (1999). Experience curves for policy making: the case of energy technologies.

[6] Moore GE (2006). Behind the ubiquitous microchip.

[7] Colpier U, Cornland D (2002) The economics of the combined cycle gas turbine-an experience curve analysis. Energy Policy : 309–322.

[8] Goldemberg J, Coelho ST, Nastari PM, Lucon O (2004) Ethanol learning curve – the Brazilian experience. Biomass and Bioenergy 26: 301–304.

[9] Schilling M (2009) Technology s-curves in renewable energy alternatives: Analysis and implications for industry and government. Energy Policy 37: 1767–1781.

[10] Energy Information Administration (2009). Annual Energy Review (AER).

[11] Zhao J (1999). The diffusion and costs of natural gas infrastructures.

[12] Maycock PD (2005) PV review: World solar PV market continues explosive growth. Refocus 6: 18–22.

[13] Nemet GF (2009) Interim monitoring of cost dynamics for publicly supported energy technologies. Energy Policy 37(3): 825-835 .

[14] Neji L, Andersen PD, Durstewitz M, Helby P, Hoppe-Kilpper M, et al. (2003). Experience curves: a tool for energy policy assessment.
